# Supplementary material for: Health and economic benefits of secondary education in the context of poverty: Evidence from Burkina Faso
Source: PLoS One. 2022 Jul 6;17(7):e0270246. doi: 10.1371/journal.pone.0270246 (PMC9258827; doi:10.1371/journal.pone.0270246)
Supplement: S1 File — (ZIP) [file pone.0270246.s001.zip › Fig S1.1.docx]

**Fig S1.1. Income analysis. Estimated increase in income per year by educational attainment level in the Boucle du Mouhoun region, Burkina Faso.**


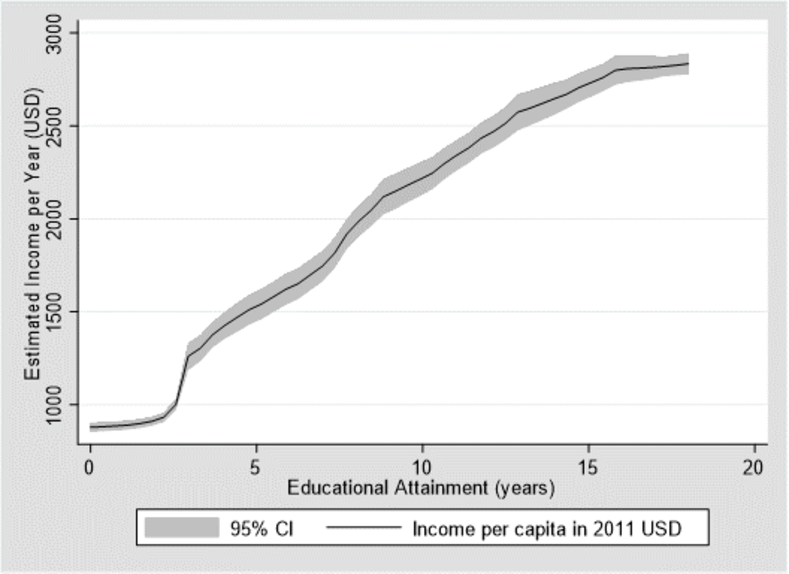


*Notes:* Estimated income by year of educational attainment based on OLS regression results from Mincer Earnings regressions, controlling for age, age squared and DHS survey year. Source: data for the Boucle du Mouhoun region using the Burkina Faso Demographic and Health Surveys (DHS) of 2003, 2010, 2014, and 2017-18 (*N*=3,924). Fig S4 in the appendix shows results for the full (nationally representative) dataset of Burkina Faso.
