# Supplementary material for: Health and economic benefits of secondary education in the context of poverty: Evidence from Burkina Faso
Source: PLoS One. 2022 Jul 6;17(7):e0270246. doi: 10.1371/journal.pone.0270246 (PMC9258827; doi:10.1371/journal.pone.0270246)
Supplement: S1 File — (ZIP) [file pone.0270246.s001.zip › Results.docx]

**Results: Relative health and financial returns to education**

**Table S1.3** summarizes overall health and income benefits of secondary education in the study area. Income gains exceeded or were almost the same as monetized life expectancy gains in 2 out of 3 proposed scenarios. Specifically, income gains exceeded monetized life expectancy gains by at least USD 3,990 in the first two scenarios for men and the first scenario for women (valuing 1 year of life 0.5 and 1 gross domestic product per capita (GDPCC)) and represented at up to more than 80% of the total benefits from secondary schooling and higher. Men seemed to have a higher relative benefit due to income gains than women. Only when valuing 1 year of life 3 GDPCC, health returns represented 56% and 74% of total returns for men and women respectively (around USD 10,000 and USD 25,000 respectively). Total benefits for women were higher than those for men ranging from around USD 12,300 to USD 33,000 compared to only USD 9,560 to USD 18,000.
