# Supplementary material for: Health and economic benefits of secondary education in the context of poverty: Evidence from Burkina Faso
Source: PLoS One. 2022 Jul 6;17(7):e0270246. doi: 10.1371/journal.pone.0270246 (PMC9258827; doi:10.1371/journal.pone.0270246)
Supplement: S1 File — (ZIP) [file pone.0270246.s001.zip › Supplementary analysis S1.docx]

**Supplementary analysis S1. Predicted incomes and relative health and financial returns to education**

*Statistical Analysis: predicted incomes and relative health and financial returns to education*

First, we estimated household income in constant 2011 international US dollar (USD) based on each household’s relative position in the wealth distribution of the country following the method outlined in Fink et al. 2017. Data on GDP per capita was obtained from the World Development Indicators [1] and data on inequality downloaded from Solt (2019). Predicted incomes were then used as outcome in standard Mincer regression models.

To compare survival benefits and earnings benefits, we first computed mean life expectancies by educational group from the HDSS data by fitting a parametric model for women and men, separately, and pooling all the three birth cohorts (1940s, 1950s and 1960s). In a second step, we estimated mean lifetime earnings using data from the latest DHS survey round (2017-2018). Based on the idea that most educational program should be completed by age 25 and can retire around age 64, we focused on ages 25-64 as “core” working period, and computed wage differentials in this age range. Given that the decision to enroll in secondary schooling is made around 15, we discounted all future earnings back to this age, assuming an average annual increase in inflation-adjusted wages of 2% and a discount rate of 3% per year [3].

For comparing life expectancy gains with income gains between secondary schooled and primary schooled, we computed three scenarios. In *Scenario 1*, we valued each additional year of life expectancy as 0.5 GDP per capita [4]; in *Scenario 2* we valued each additional year of life expectancy as 1 GDP per capita; and, lastly, in *Scenario 3*, we valued each additional year of life expectancy as 3 GDP per capita [5]. GDP per capita used is from year 2017 (1696.5 constant 2011 international USD) taken from the World Development Indicators [1].

*Results: Income benefits from earnings regressions*

**Fig S1.1** shows the basic relationship between schooling and household incomes. **Table S1.1** shows the results for the earning regressions. On average, secondary or higher schooling was associated with an income increase of 23% (Standard Error [SE] 0.03; CI 0.17 - 0.29) compared to primary schooling in urban settings, and 64% (SE 0.04; CI 0.57 - 0.72) in the pooled sample. Returns for women were 5% points higher than male returns: 33% (SE 0.05; CI 0.23 - 0.42) vs. 28% (SE 0.04; CI 0.20 - 0.36). The difference between rural and urban residents was even higher with more than 20% points difference: 44% (SE 0.08; CI 0.29 - 0.59) vs. 23% (SE 0.03; CI 0.17 - 0.29). In terms of predicted lifetime earnings, secondary- and higher-schooled women gained USD 8,149 (CI 6,297 - 10,116) and men USD 7,875 (CI 6,085 - 9,777) more in a lifetime than people who only attended primary school (**Table S1.2**). Mean lifetime earnings for secondary- and higher-schooled were USD 30,401 for men and USD 31,456 for women compared to only USD 22,526 and USD 23,307 for individuals who attended only 1 to 6 years of formal education.

**References**

1. World Bank. World Development Indicators; 2020 [cited 2020 May 9]. Available from: URL: https://databank.worldbank.org/source/world-development-indicators.

2. Solt F. The Standardized World Income Inequality Database, Version 8; 2019 [cited 2020 May 9]. Available from: URL: https://dataverse.harvard.edu/dataset.xhtml?persistentId=doi:10.7910/DVN/LM4OWF.

3. Drummond M, Sculpher MJ, Claxton K, Stoddart GL, Torrance GW. Methods for the economic evaluation of health care programmes. 4th ed. Oxford: Oxford University Press; 2015.

4. Woods B, Revill P, Sculpher M, Claxton K. Country-Level Cost-Effectiveness Thresholds: Initial Estimates and the Need for Further Research. Value Health 2016; 19(8):929–35.

5. Marseille E, Larson B, Kazi DS, Kahn JG, Rosen S. Thresholds for the cost-effectiveness of interventions: Alternative approaches. Bulletin of the World Health Organization 2015; 93(2):118–24.
