# Supplementary material for: Health and economic benefits of secondary education in the context of poverty: Evidence from Burkina Faso
Source: PLoS One. 2022 Jul 6;17(7):e0270246. doi: 10.1371/journal.pone.0270246 (PMC9258827; doi:10.1371/journal.pone.0270246)
Supplement: S1 File — (ZIP) [file pone.0270246.s001.zip › Table S1.1.docx]

**Table S1.1. Income analysis: Results from OLS earnings regression models for the Boucle du Mouhoun region, Burkina Faso.**

|  |  | **Basic Model** |  | **Stratified by Sex** | |  | **Stratified by Type of Place of Residence** | |
| --- | --- | --- | --- | --- | --- | --- | --- | --- |
| **Variable** |  |  |  | Males | Females |  | Urban | Rural |
|  |  |  |  |  |  |  |  |  |
| Age | | 0.02** |  | 0.04*** | 0.02* |  | 0 | 0.03*** |
|  |  | (0.01; 0.00 - 0.03) |  | (0.01; 0.02 - 0.05) | (0.01; -0.00 - 0.04) |  | (0.01; -0.02 - 0.02) | (0.01; 0.02 - 0.05) |
| Educational Attainment | |  |  |  |  |  |  |  |
| No schooling | | -0.49*** |  | -0.26*** | -0.29*** |  | -0.20*** | -0.14*** |
|  |  | (0.03; -0.56 - -0.43) |  | (0.04; -0.33 - -0.19) | (0.04; -0.37 - -0.22) |  | (0.03; -0.27 - -0.14) | (0.04; -0.21 - -0.07) |
| Primary schooling | | Reference group | | | | | | |
| Secondary or higher | | 0.64*** |  | 0.28*** | 0.33*** |  | 0.23*** | 0.44*** |
|  |  | (0.04; 0.57 - 0.72) |  | (0.04; 0.20 - 0.36) | (0.05; 0.23 - 0.42) |  | (0.03; 0.17 - 0.29) | (0.08; 0.29 - 0.59) |
| Observations | | 3,924 |  | 1,499 | 2,425 |  | 1,449 | 2,475 |
| R-squared |  | 0.264 |  | 0.591 | 0.453 |  | 0.374 | 0.052 |

*Notes:* A total of 3,924 individuals were surveyed in 2003, 2010, 2014, and 2017-18. The dependent variable was Ln (Annual income) estimated in constant 2011 international US Dollar (USD). Coefficients represent the yearly increase in income on a natural logarithmic scale. Robust standard errors (SE) and 95% Confidence Interval (CI) in parentheses (SE; CI). *** p<0.01, ** p<0.05, * p<0.1. In all models we controlled for age squared; in the stratified models we additionally controlled for survey round. OLS: ordinary least squares.
