# Supplementary material for: Health and economic benefits of secondary education in the context of poverty: Evidence from Burkina Faso
Source: PLoS One. 2022 Jul 6;17(7):e0270246. doi: 10.1371/journal.pone.0270246 (PMC9258827; doi:10.1371/journal.pone.0270246)
Supplement: S1 File — (ZIP) [file pone.0270246.s001.zip › Table S1.2.docx]

**Table S1.2. Lifetime earnings in constant 2011 international US Dollar, separately by sex and educational level, in the Boucle du Mouhoun region, Burkina Faso (2017-2018).**

|  |  |  | **Male** | |  | **Female** | |
| --- | --- | --- | --- | --- | --- | --- | --- |
|  |  |  |  |  |  |  |  |
|  |  |  | Lifetime earnings | (95% CI) |  | Lifetime earnings | (95% CI) |
| Highest schooling attainment | | |  |  |  |  |  |
| none (0 years) | |  | 17,028 | (16,177 - 17,924) |  | 17,619 | (16,738 - 18,546) |
| primary (1 - 6 years) | | | 22,526 | (20,736 - 24,428) |  | 23,307 | (21,455 - 25,274) |
| secondary (7+ years) | | | 30,401 | (28,611 - 32,303) |  | 31,456 | (29,604 - 33,423) |
|  |  |  |  |  |  |  |  |

*Notes:* Earnings were predicted using data from the Boucle du Mouhoun region, Burkina Faso. Lifetime earnings were calculated from 25 to 64 years with a discounting factor of 1.01 per year starting discounting from 15 years on (annual growth in real wages: 2%, annual discount rate: 3%). 95% Confidence Interval [CI] are given in brackets. Source: Boucle du Mouhoun region data, Burkina Faso DHS survey 2017-18.
