# Supplementary material for: Health and economic benefits of secondary education in the context of poverty: Evidence from Burkina Faso
Source: PLoS One. 2022 Jul 6;17(7):e0270246. doi: 10.1371/journal.pone.0270246 (PMC9258827; doi:10.1371/journal.pone.0270246)
Supplement: S1 File — (ZIP) [file pone.0270246.s001.zip › Table S1.3.docx]

**Table S1.3. Comparison of gains in monetized life expectancy and lifetime** **earnings between secondary and higher- and primary-schooled (USD).**

|  | **Male** | | | | |  | **Female** | | | | |
| --- | --- | --- | --- | --- | --- | --- | --- | --- | --- | --- | --- |
|  |  |  |  |  |  |  |  |  |  |  |  |
|  | ∆MLE | ∆LTE | Total Benefits | % of total Benefits due to Health | |  | ∆MLE | ∆LTE | Total Benefits | % of total Benefits due to Health | |
|  |  |  |  |  |  |  |  |  |  |  |  |
| Scenario 1 | 1,688 | 7,875 | 9,564 | 17.65% | |  | 4,158 | 8,149 | 12,307 | 33.79% | |
| Scenario 2 | 3,376 | 7,875 | 11,252 | 30.01% | |  | 8,316 | 8,149 | 16,465 | 50.51% | |
| Scenario 3 | 10,129 | 7,875 | 18,004 | 56.26% | |  | 24,949 | 8,149 | 33,098 | 75.38% | |
|  |  |  |  |  |  |  |  |  |  |  |  |

*Notes:* MLE: monetized life expectancy equivalent, LTE: lifetime earnings. Scenario 1: 1 year of life expectancy equals 0.5 gross domestic product per capita (GDPCC), Scenario 2: 1 year of life expectancy equals 1 GDPCC, Scenario 3: 1 year of life expectancy equals 3 GDPCC.
