# Supplementary material for: Health and economic benefits of secondary education in the context of poverty: Evidence from Burkina Faso
Source: PLoS One. 2022 Jul 6;17(7):e0270246. doi: 10.1371/journal.pone.0270246 (PMC9258827; doi:10.1371/journal.pone.0270246)
Supplement: S1 Fig — (DOCX) [file pone.0270246.s002.docx]

## Fig S1. Study participant flow diagram DHS sample Boucle du Mouhoun region.


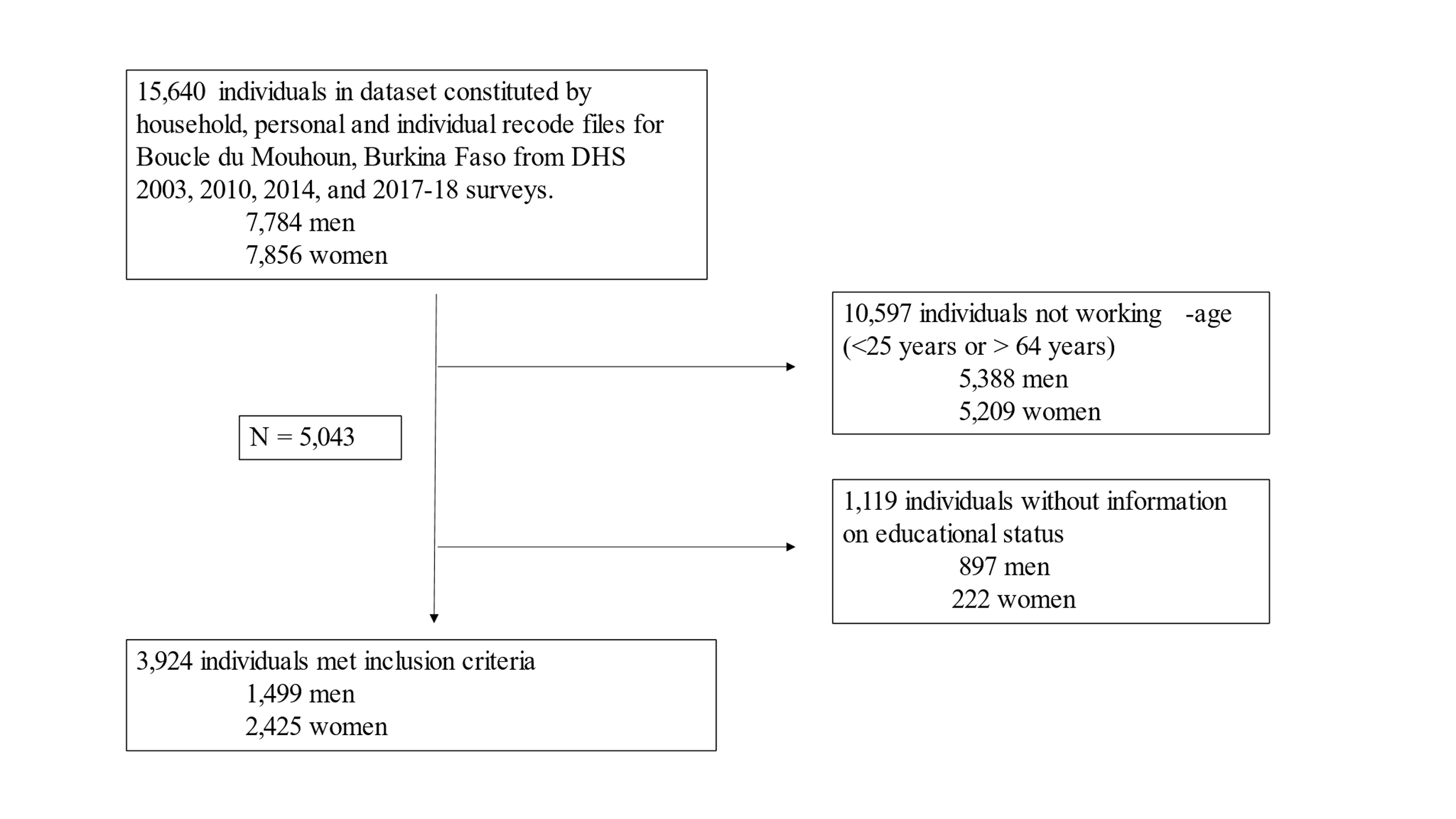


*Notes:* Individuals were surveyed in the Burkina Faso Demographic and Health Surveys (DHS) of 2003, 2010, 2014, and 2017-18.
