# Supplementary material for: Health and economic benefits of secondary education in the context of poverty: Evidence from Burkina Faso
Source: PLoS One. 2022 Jul 6;17(7):e0270246. doi: 10.1371/journal.pone.0270246 (PMC9258827; doi:10.1371/journal.pone.0270246)
Supplement: S2 Fig — (DOCX) [file pone.0270246.s003.docx]

## Fig S2. Framework of statistical analysis.


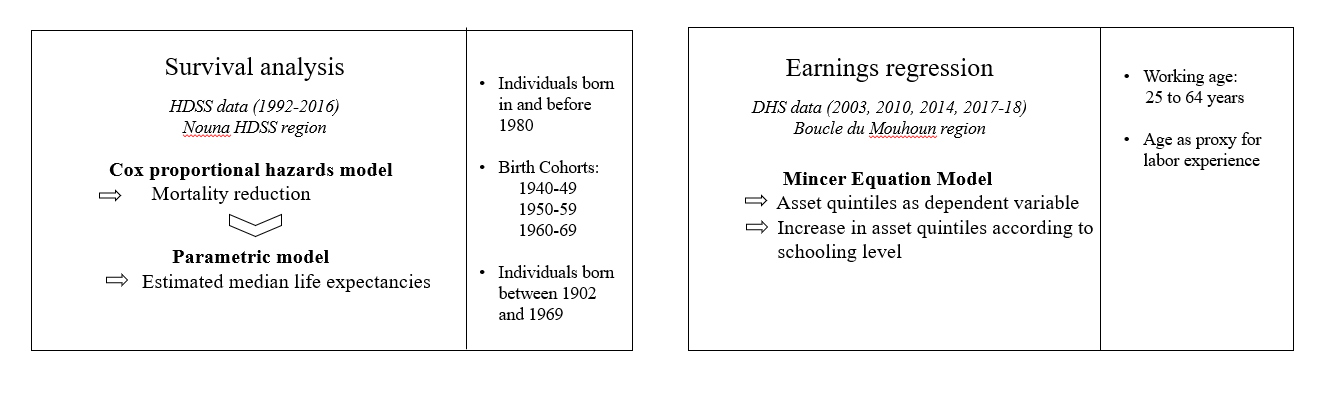


*Notes:* The graphic shows an overview over the approach used for statistical analysis of the Burkina Faso DHS data and Nouna HDSS data.
