# Supplementary material for: Health and economic benefits of secondary education in the context of poverty: Evidence from Burkina Faso
Source: PLoS One. 2022 Jul 6;17(7):e0270246. doi: 10.1371/journal.pone.0270246 (PMC9258827; doi:10.1371/journal.pone.0270246)
Supplement: S3 Fig — (DOCX) [file pone.0270246.s004.docx]

## Fig S3. Survival according to schooling - Kaplan Meier Graphs.


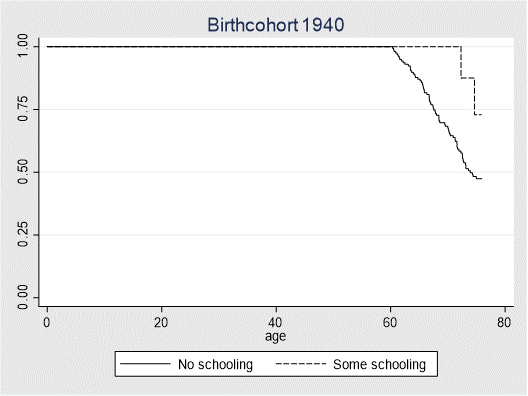


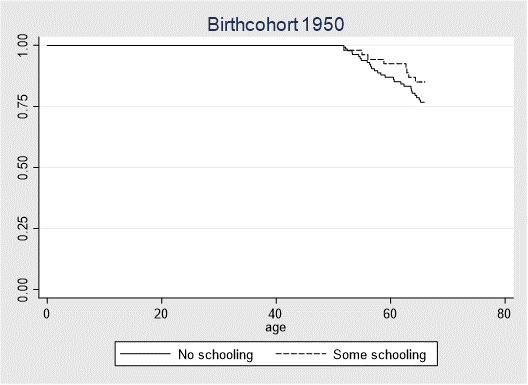


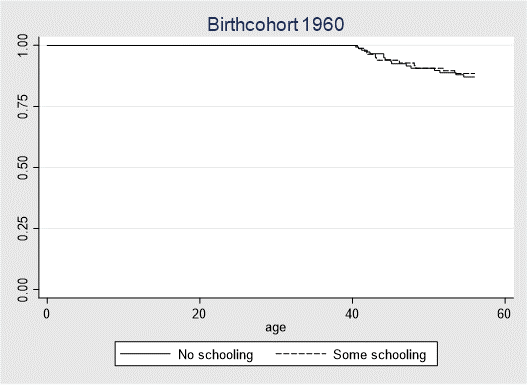


*Notes:* Survival curves based on regression results from the Cox-Proportional Hazards Model of a total of 187, 237 and 305 individuals in the 1940s, 1950s and 1960s cohort surveyed from 1992 to 2016 in the Nouna HDSS, respectively. Individuals from the 1940s, 1950s and 1960s cohort were observed from ages (in years) 52, 42 and 32 on, respectively.
