# Supplementary material for: Health and economic benefits of secondary education in the context of poverty: Evidence from Burkina Faso
Source: PLoS One. 2022 Jul 6;17(7):e0270246. doi: 10.1371/journal.pone.0270246 (PMC9258827; doi:10.1371/journal.pone.0270246)
Supplement: S4 Fig — (DOCX) [file pone.0270246.s005.docx]

## Fig S4. Wealth analysis: Estimated increase in asset quintiles by educational attainment for entire Burkina Faso.

*Notes:* Asset quintile by year of educational attainment based on OLS regression results from Mincer Earnings regressions, controlling for age and DHS survey year. Source: data for entire Burkina Faso using the Burkina Faso Demographic and Health Surveys (DHS) of 2003, 2010, 2014, and 2017-18 (*N*=54,540).
