# Supplementary material for: Health and economic benefits of secondary education in the context of poverty: Evidence from Burkina Faso
Source: PLoS One. 2022 Jul 6;17(7):e0270246. doi: 10.1371/journal.pone.0270246 (PMC9258827; doi:10.1371/journal.pone.0270246)
Supplement: S1 Table — (DOCX) [file pone.0270246.s006.docx]

## Table S1. Missingness table Nouna HDSS.

| **Variable** | **Missing (N)** | **Total** | **Missing (%)** |
| --- | --- | --- | --- |
|  |  |  |  |
| Age* | 0 | 49,865 | 0.00 |
| Sex | 0 | 49,865 | 0.00 |
| Educational attainment category** | 34,973 | 49,865 | 70.14 |
| male | 15,690 | 23,451 | 44.86 |
| female | 19,283 | 26,414 | 55.14 |
|  |  |  |  |
| urban | 6,685 | 14,963 | 19.11 |
| rural | 28,288 | 34,902 | 80.89 |
|  |  |  |  |
| Survival status | 0 | 49,865 | 0.00 |
|  |  |  |  |
| Complete cases |  | 14,892 |  |

*Notes:* 49,865 individuals from the dataset were (former) residents of the Nouna HDSS (in the period 1992 to 2016), were observed for at least 1 year and born in or before 1980. The complete case dataset included 14,892 individuals. *Age of entry and age of exit. **The last educational attainment given defined the educational attainment group.
