# Supplementary material for: Health and economic benefits of secondary education in the context of poverty: Evidence from Burkina Faso
Source: PLoS One. 2022 Jul 6;17(7):e0270246. doi: 10.1371/journal.pone.0270246 (PMC9258827; doi:10.1371/journal.pone.0270246)
Supplement: S3 Table — (DOCX) [file pone.0270246.s008.docx]

## Table S3. Sample characteristics of cohorts followed over time.

|  | **Person-years** | **% of person-years** | **Deaths (cases)** | **Incidence rate (per 100 py)** | |  |
| --- | --- | --- | --- | --- | --- | --- |
| *Cohort born 1940-49* | | | | | |  |
|  |  |  |  |  |  |  |
| All | 18,081 | 100.00 | 370 | 2.05 | (1.85 - 2.27) |  |
| Highest schooling attainment | |  |  |  |  |  |
| none (0 years) | 14,982 | 82.86 | 318 | 2.12 | (1.90 - 2.37) |  |
| primary (1 - 6 years) | 2,327 | 12.87 | 33 | 1.42 | (1.01 - 1.99) |  |
| secondary (7+ years) | 0,771 | 4.26 | 19 | 2.46 | (1.57 - 3.86) |  |
|  |  |  |  |  |  |  |
| *Cohort born 1950-59* | | | | | |  |
|  |  |  |  |  |  |  |
| All | 27,057 | 100.00 | 265 | 0.98 | (0.87 - 1.10) |  |
| Highest schooling attainment | |  |  |  |  |  |
| none (0 years) | 16,238 | 60.02 | 174 | 1.07 | (0.92 - 1.24) |  |
| primary (1 - 6 years) | 8,826 | 32.62 | 76 | 0.86 | (0.69 - 1.08) |  |
| secondary (7+ years) | 1,992 | 7.36 | 15 | 0.75 | (0.45 - 1.25) |  |
|  |  |  |  |  |  |  |
| *Cohort born 1960-69* | | | | | |  |
|  |  |  |  |  |  |  |
| All | 41,369 | 100.00 | 203 | 0.49 | (0.43 - 0.56) |  |
| Highest schooling attainment | |  |  |  |  |  |
| none (0 years) | 23,997 | 58.01 | 122 | 0.51 | (0.43 - 0.61) |  |
| primary (1 - 6 years) | 13,369 | 32.32 | 64 | 0.48 | (0.37 - 0.61) |  |
| secondary (7+ years) | 4,003 | 9.68 | 17 | 0.42 | (0.26 - 0.68) |  |
|  |  |  |  |  |  |  |

*Note:* Sample of the three cohorts included 6,634 individuals followed over the years 1992 - 2016. Data are from the Nouna Health and Demographic and Surveillance (HDSS), Burkina Faso.
