# Supplementary material for: Health and economic benefits of secondary education in the context of poverty: Evidence from Burkina Faso
Source: PLoS One. 2022 Jul 6;17(7):e0270246. doi: 10.1371/journal.pone.0270246 (PMC9258827; doi:10.1371/journal.pone.0270246)
Supplement: S4 Table — (DOCX) [file pone.0270246.s009.docx]

## Table S4. Wealth regressions for entire Burkina Faso with education as categorized variable.

*Notes:* A total of 54,540 individuals were surveyed in 2003, 2010, 2014, and 2017-18. The dependent variable was Ln (Asset Quintile). Coefficients represent the yearly increase in asset holdings on a natural logarithmic scale. Robust standard errors (SE) and 95% Confidence Interval (CI) in parentheses (SE; CI). *** p<0.01, ** p<0.05, * p<0.1. In all models we controlled for age squared; in the stratified models we additionally controlled for survey round. OLS: ordinary least squares.
