# Supplementary material for: Health and economic benefits of secondary education in the context of poverty: Evidence from Burkina Faso
Source: PLoS One. 2022 Jul 6;17(7):e0270246. doi: 10.1371/journal.pone.0270246 (PMC9258827; doi:10.1371/journal.pone.0270246)
Supplement: Attachment — Submitted filename: PLOS_One_D_21_01735_Review.pdf [file pone.0270246.s011.pdf]

Review of "Health and economic benefits of secondary education"

This paper provides evidence about the health and economic benefits of secondary education in rural Burkina Faso, using two sources of evidence: long-run health surveillance data, and the DHS. They find evidence that attending secondary school is associated with higher income as well as higher life expectancy.

My overriding comment about this paper is that it provides only correlational evidence about the relationship of interest, when we know that correlational evidence is probably severely limited, and causal evidence is widely available. The authors' acknowledgment of this fact (absence of causal evidence) is limited to a single sentence in the limitations section. Particularly for the analysis of the returns to education, there is an absolutely enormous economics literature analyzing causal effects of education on earnings in the developing world already published, and so the bar for an additional contribution is high. The authors fail to cite the majority of this literature, I won't aim to summarize it myself but will refer the authors to two recent literature reviews: Peet et al. in the Economics of Education Review 2020, and Patrinos and Psacharopoulos in the Economics of Education (also 2020). I am (furthermore) additionally skeptical of this analysis because the authors don't have actual income data, but are imputing income based on assets as reported in the DHS.

My suggestion is that this entire analysis be dropped from the paper and that the authors focus only on the health benefits of secondary education. Here the authors seem to be on stronger ground in reporting only a correlation for several reasons: they are using a relatively novel data source with long-term longitudinal data; and the literature is smaller. However, the authors again need to do a lot more to situate their findings in this literature, however large it is. There are a number of papers cited (citations 3 to 10, broadly), but in the discussion section the authors should more clearly elaborate on what they find relative to the existing papers and what contribution these findings make. Are the existing estimates from comparable settings or using similar research designs, or are they different? Is the magnitude of the relationship between education and mortality they estimate similar to existing estimates, or different? Is the potential bias due to endogenous selection into education similar in this analysis, or worse? (In general - in a context such as rural Burkina Faso where almost no one attends secondary school, we would expect this bias to be particularly acute: only highly motivated and intelligent students from families with adequate resources and/or a high interest in education would attend secondary school.)

Having dropped the analysis of income returns to education, the authors would then have more space for a longer discussion section that might fruitfully grapple with some of these questions around situating the mortality analysis in the literature and unpacking how to interpret this relationship.
