# Supplementary material for: Health and economic benefits of secondary education in the context of poverty: Evidence from Burkina Faso
Source: PLoS One. 2022 Jul 6;17(7):e0270246. doi: 10.1371/journal.pone.0270246 (PMC9258827; doi:10.1371/journal.pone.0270246)
Supplement: Attachment — Submitted filename: Response to Reviewers_24May2022.docx [file pone.0270246.s013.docx]

# Response to Reviewers

Review Comments to the Author

1) I have now been asked to serve as a guest editor for this submission; in the interests of transparency, I should note I was previously serving as a reviewer (reviewer #2). I reviewed the updated materials you provided and also invited reviewer #1 to review the manuscript again; s/he declined to do so. My judgment is that you have responded thoroughly to this reviewer's comments. Following up on my own comments, I would like to request some minor additional revisions.

*We thank the reviewer for his/her judgment of this paper and the work invested in a second revision.*

2) I am uncomfortable with the use of imputed income in an analysis of returns to education, though of course you are right that the DHS does not report income. I suggest that you conduct this analysis primarily using an asset index drawing on the asset information that is actually reported in the DHS (i.e., estimate the correlation between education and assets.) Since this is not in any case a causal analysis that would fall into the core returns to education literature, it does not seem necessary to use an artificial income measure. If you wish to also include the analysis using imputed income as an extension in the paper, of course you can, but I suggest this be a secondary analysis.

*We replaced the income estimates with the original asset score in form of asset quintiles and used ln(asset quintile) as dependent variable to calculate increase in asset holdings and described health and economic benefits separately.*

*“We use asset ownership as a proxy for permanent income and estimate average increases in asset holdings associated with additional education. Asset quintiles were used as outcome in standard Mincer regression models to estimate returns to education (38–40).”*

*We moved the predicted income table to the supporting information (****Supplementary Analysis S1****) as suggested.*

*“For methodology and estimation of predicted incomes and relative health and financial returns see* ***Supplementary Analysis S1*** *in the supporting information.”*

3) In the discussion section, you focus primarily on comparing your estimate of the returns to education to other estimates. What about other estimates of mortality benefits of education - are there any such estimates? What is the magnitude of those estimates compared to yours? It may be that the literature here is minimal; if so, you can just note this.

*There is indeed not nearly as much literature on the survival returns to education - we have now added the two main references to the revised Discussion section, where we write:*

*“Concerning the magnitude of health benefits, Gathmann, Jürges et al. (2015) estimate a 2.0% reduction in 20-year-mortality rate for only men (9) and Lleras-Muney (2005) a 10-year mortality rate reduction by 1.3 to 3.6% per additional year of education (47). Those results are similar to our estimations finding a 3.6% reduction in the mortality hazard.”*

4) Following up on your response to the previous referee report: it is not necessarily the case that bias in the estimated returns to education due to selection into education is the same across the two dimensions examined (income and mortality). For example, if very intelligent individuals (who are not necessarily healthy or rich) select into education, the selection bias may be larger for income vis-à-vis mortality. If individuals from wealthy families select into education, then the bias may be inverted (larger for mortality, since wealthier families live longer, ceteris paribus). It is up to you whether you wish to engage with this point in the discussion section, but it may be useful context for your interpretation.

*We thank the reviewer for bringing this to our attention; we have now added the following point to the revised Discussion section:*

*“Fifth, endogenous selection bias cannot be excluded: Wealthy individuals for example might select into education and bias health and income benefits positively, or very intelligent individuals who might bias stronger income than mortality results.”*
